# Theory for Perfect Transmodal Fabry-Perot Interferometer

**DOI:** 10.1038/s41598-017-18408-5

**Published:** 2018-01-08

**Authors:** Xiongwei Yang, Joshua M. Kweun, Yoon Young Kim

**Affiliations:** 1 BK21 Plus Transformative Program for Creative Mechanical & Aerospace Engineers, Seoul National University, 1 Gwanak-ro, Gwanak-gu, Seoul, 08826 South Korea; 2 School of Mechanical and Aerospace Engineering, Seoul National University, 1 Gwanak-ro, Gwanak-gu, Seoul, 08826 South Korea; 3 Institute of Advanced Machines and Design, Seoul National University, 1 Gwanak-ro, Gwanak-gu, Seoul, 08826 South Korea

## Abstract

We establish the theory for *perfect* transmodal Fabry-Perot interferometers that can convert longitudinal modes solely to transverse modes and vice versa, reaching up to 100% efficiency. Two exact conditions are derived for plane mechanical waves: simultaneous constructive interferences of each of two coupled orthogonal modes, and intermodal interference at the entrance and exit sides of the interferometer with specific skew polarizations. Because the multimodal interferences and specific skew motions r**eq**uire unique anisotropic interferometers, they are realized by metamaterials. The observed peak patterns by the transmodal interferometers are similar to those found in the single-mode Fabry-Perot resonance, but multimodality complicates the involved mechanics. We provide their design principle and experimented with a fabricated interferometer. This theory expands the classical Fabry-Perot resonance to the realm of mode-coupled waves, having profound impact on general wave manipulation. The transmodal interferometer could sever as a device to transfer wave energy freely between dissimilar modes.

## Introduction

Wave mode governs energy propagation pattern, and thus modal interactions by mode conversion have been widely explored in electromagnetics to acoustics^1–14^ for nonreciprocal propagation^1^, energy channeling in magnetic fusion^6^, cloaking^9,10^ and others. For instance, the elastic wave mode conversion can be used to generate transverse modes from longitudinal waves for various ultrasonic applications such as non-destructive and medical tests^15,16^. The common mode conversion methods use Snell’s critical angle^17^. However, these methods can hardly produce high mode-converting efficiency due to impedance mismatch. Although complete mode conversion might be theoretically possible with double negative metamaterials^18,19^, their use is very restrictive and no actual realization is attempted. Recently, a mode conversion phenomenon through anisotropic slabs has been reported and the observed phenomenon was called the TFPR (transmodal Fabry-Perot resonance)^20^. On the assumption that the slabs are weekly-coupled and the background mediums are low-reflective, the pattern of local peaks in the mode conversion transmission was observed. Due to the assumptions, however, the perfect mode-conversion showing exactly the same interference phenomenon as observed in the classical Fabry-Perot interference phenomenon was not possible.

## Results

Here, we establish the exact theory of *perfect* TFPR without any assumptions or approximations. With this theory, the mechanism of the perfect mode conversion having full or maximal transmission can be theoretically explained and accordingly, a device of the perfect transmodal Fabry-Perot interferometers (TFPIs) can be engineered. We realize the perfect TFPI through elaborately designing anisotropic mechanical metamaterials, because metamaterials can realize extraordinary material properties needed to manipulate waves in an unprecedented manner (see, e.g.,^21–25^).

**Figure 1:**
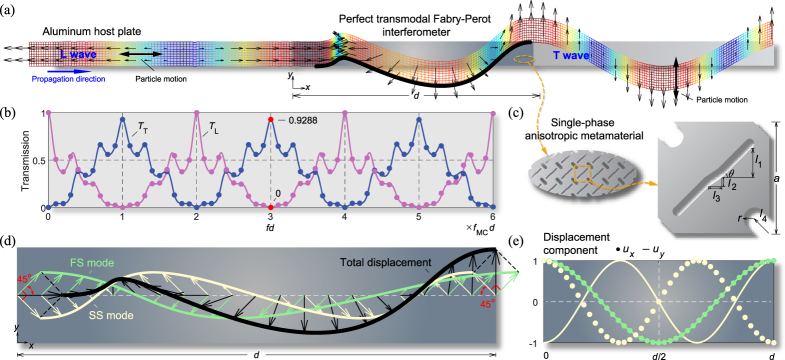
Perfect mode conversion for a normally incident L wave. (**a**) Snapshot of the transient displacement field at the fundamental PMC frequency of *f*_MC_. An incident longitudinal wave travelling into the mode-converting interferometer between an aluminum host plate (Young’s modulus *E*_Al_ = 71 GPa, density *ρ*_Al_ = 2700 kg/m^3^, Poisson’s ratio *ν*_Al_ = 0.33) is perfectly converted to a transverse wave upon exiting the interferometer. The prescribed horizontal displacement (*u*_*x*_) at *x* = 0 is *u*_0_sin(2π*f*_MC_ *t*), and the snapshot is taken at the time *t* = 29.25/*f*_MC_. (*f*_MC_ *d* = 2.87 kHz·m) (see Supplementary Movie S1). The thick black line indicates the total displacement of the interferometer. (**b**) The transmission ratios *T*_L_ (L-to-L transmission) and *T*_T_ (L-to-T transmission), calculated by theoretical analysis (lines) and full-wave numerical simulation (dots). (**c**) The unit cell of the interferometer, with the effective stiffness constants *C*_11_ = 12.983 GPa, *C*_66_ = 13.053 GPa and *C*_16_ = 5.006 GPa, and the effective mass density *ρ* = 2193 kg/m^3^. These values satisfy the exact conditions for PMC, as expressed in Eqs (1, 2). The unit cell has elaborately designed slits (*θ* = 45°, *l*_1_ = 0.2636*a*, *l*_2_ = 0.0800*a*, *l*_3_ = 0.1200*a*, *l*_4_ = 0.1850*a*, *r* = 0.1000*a*, *a* = *d*/100) machined onto the aluminum host plate. (**d**) Schematic demonstration of the mode conversion mechanism using two L-T coupled wave modes inside the interferometer, denoted by the fast skew mode (FS) with *d* = *λ*_FS_ and the slow skew mode (SS) with *d* = 3/2*λ*_SS_. (**e**) Distribution of the horizontal (*u*_*x*_) and vertical (*u*_*y*_) displacements for the FS and SS modes inside the interferometer.

For this investigation, we consider plane mechanical waves consisting of longitudinal (L) and transverse (T) modes propagating in the horizontal or *x* direction. The L mode is a dilatational mode with horizontal polarization and the T mode is a shear mode with vertical polarization propagating along the horizontal axis. Figure 1a shows the TFPI that perfectly converts an incident L mode into a transmitted T mode. Perfect mode conversion (PMC) implies that the L mode is perfectly and maximally converted to a T mode *without transmitting any of the L mode*. Figure 1b shows how PMC occurs at distinct frequencies, *f* = *f*_MC_, 3*f*_MC_, 5*f*_MC_, … etc., in a repeated resonance pattern. At these frequencies, *T*_T_ is always maximized and *T*_L_ simultaneously disappears; *T*_T_ (*T*_L_) represents the transmission ratio of the transmitted T (L) mode power to the incident L mode power shown in Fig. 1a. The TFPI is designed by an anisotropic metamaterial, the unit cell of which is depicted in Fig. 1c.

To explain the PMC mechanism from the L to T modes or from the T to L modes, wave analysis using a theoretical model was performed (see the supplementary file for further details). Figure 1d shows that two eigenmodes of the TFPI are formed by the constructive interferences of the corresponding propagating waves in the +*x* and −*x* directions at the PMC frequency *f* = *f*_MC_. However, their interference patterns are different. At the same time, these waves have particular mode shapes of skewed polarization, as shown in Fig. 1e. Based on their phase speeds, we call these modes the fast skew (FS) and slow skew (SS) modes.

The theory for the perfect TFPI can be described by the following exact conditions (see the supplementary file for the derivations):

1.a $$d={N}_{{\rm{FS}}}\frac{{\lambda }_{{\rm{FS}}}}{2},{N}_{{\rm{FS}}}=m\cdot {n}_{{\rm{FS}}}\quad (m=1,3,5,\ldots ),$$

1.b $$d={N}_{{\rm{SS}}}\frac{{\lambda }_{{\rm{SS}}}}{2},{N}_{{\rm{SS}}}=m\cdot {n}_{{\rm{SS}}}\quad (m=1,3,5,\ldots ),$$

1.c $${n}_{{\rm{FS}}}+{n}_{{\rm{SS}}}=odd({n}_{FS} < {n}_{SS}:{\rm{coprime}}\,{\rm{integers}})$$

In addition, it is required that

2 $${C}_{11}={C}_{66}.$$

To interpret the physics involved in Eqs (1, 2), we start with Eqs (1.a, b). They represent the constructive interferences of each of the skew coupled modes in the interferometer, but the simultaneous constructive interferences of the two coupled modes are required unlike the classical Fabry-Perot interference^26,27^ involving only a single mode. The physical significance of Eq. (1.c) is that the SS mode interference is coupled with the FS mode interference with specific phase difference; this is unique in the transmodal interference not appearing in the classical Fabry-Perot interference. Eq. (2) ensures that the orientation of the skew motion aligns with an angle of ±45^o^.

We can show that due to the conditions (1) and (2), the FS and SS modes (see Fig. 1e) must be so related as to satisfy the relations that ${u}_{x}^{{\rm{FS}}}{|}_{x=0}={u}_{x}^{{\rm{FS}}}{|}_{x=d}$,$\,{u}_{y}^{{\rm{FS}}}{|}_{x=0}={u}_{y}^{{\rm{FS}}}{|}_{x=d}$ and ${u}_{x}^{{\rm{SS}}}{|}_{x=0}=-{u}_{x}^{{\rm{SS}}}{|}_{x=d}$,$\,{u}_{y}^{{\rm{SS}}}{|}_{x=0}=-{u}_{y}^{{\rm{SS}}}{|}_{x=d}$ at the PMC frequencies (*f*_MC_, 3*f*_MC_, …), where *u*_*x*_ and *u*_*y*_ denote the *x*- and *y*-direction displacements. Waves at the entrance and exit interfaces of the interferometer are denoted by the subscripts *x* = 0 and *x* = *d*, respectively. The implication of the relations is that the two eigenmodes in the interferometer of size *d* are all phase-matched over *d*. Furthermore, the phase change for one mode (FS mode in this case) is 2*π* and that for the other mode (SS mode) is 2*π* + *π*. The skew motions of the two modes show that the FS mode oscillates along an angel of +45^o^ or −135^o^ with respect to the *x* axis, whereas the SS mode oscillates along an angel of −45^o^ or +135^o^. Because the skew motions take place along ± 45^o^, the following relations hold true: ${u}_{x}^{{\rm{FS}}}=c{u}_{x}^{{\rm{SS}}}$ and ${u}_{y}^{{\rm{FS}}}=-c{u}_{y}^{{\rm{SS}}}$ at *x* = 0; ${u}_{x}^{{\rm{FS}}}=-c{u}_{x}^{{\rm{SS}}}$ and ${u}_{y}^{{\rm{FS}}}=c{u}_{y}^{{\rm{SS}}}$ at *x* = *d* with *c* = ±1. The symbol *c* denotes the relative participation ratio of the FS mode to the SS mode. If *c* = 1, the superposition of the two skew modes yields: (*u*_*x*_ ≠ 0, *u*_*y*_ = 0) at *x* = 0 and (*u*_*y*_ ≠ 0, *u*_*x*_ = 0) at *x* = *d*. Therefore, perfect L-to-T mode conversion is possible, as shown in Fig. 1(a,b). If *c* = −1, perfect T-to-L mode conversion is possible; some examples of T-to-L conversions are given in the supplementary file.

If we rewrite Eq. (1) as:

3.1 $${\rm{\Gamma }}\triangleq \frac{{C}_{11}+{C}_{66}}{2}=\frac{{C}_{{\rm{MC}}}}{2}(\frac{1}{{n}_{{\rm{FS}}}^{2}}+\frac{1}{{n}_{{\rm{SS}}}^{2}}),$$

3.2 $${\rm{\Pi }}\triangleq \sqrt{{C}_{11}{C}_{66}-{C}_{16}^{2}}=\frac{{C}_{{\rm{MC}}}}{{n}_{{\rm{FS}}}{n}_{{\rm{SS}}}},{C}_{{\rm{MC}}}\triangleq 4\rho {f}_{{\rm{MC}}}^{2}{d}^{2},$$

insights into the material requirements for the perfect TFPI are attained. The effective material properties of the interferometer cannot be arbitrary but must satisfy the conditions given in Eqs (2, 3); this is quite different from the classical Fabry-Perot interferometers. At *fd* = *f*_MC_ *d* = 2.87 kHz where the PMC peak appears (Fig. 1b), the unit cell of the interferometer shown in Fig. 1c exactly satisfies the conditions in Eqs (2, 3) for the selected values of *N*_FS_ *=* *n*_FS_ = 2 and *N*_SS_ *=* *n*_SS_ = 3. A detailed procedure to design the corresponding metamaterial is given in the supplementary file. As long as the long wavelength assumption at the PMC frequencies is valid, the effective properties of a metamaterial can be accurately evaluated by static limit through static homogenization^28^.

Further analysis shows that the transmission ratio at *f* = *mf*_MC_ (*m* = 1, 3, …) can be analytically obtained,

4 $${T}_{{\rm{T}}}=\xi {\{\frac{\xi +1}{2\xi }[1-{(\frac{1-\xi }{1+\xi })}^{2}]\}}^{2},{T}_{{\rm{L}}}=0,\xi \triangleq \sqrt{\frac{1-{\nu }_{0}}{2}}.$$

Eq. (4) shows that while *T*_L_ becomes identically zero, *T*_T_ can be 100% if *ν*_0_ → −1 (*ν*_0_ is Poisson’s ratio of the base isotropic medium). Our analysis was based on a plane stress condition that complies with thin plates. The results for the plane strain case are given in the supplementary file. In Eq. (4), ξ can be interpreted as the mechanical impedance ratio between the L and T waves in the base medium. If *ξ* = 1, PMC with 100% transmission is achieved. Therefore, *T*_T_ can be 100% if *c*_11_ = *c*_66_ and *c*_16_ = 0 where *c*_*ij*_ is the stiffness coefficients of the base medium. For the aluminum base material used in this work, *ξ* = 0.5788. Although we consider mode conversion from an L to a T wave in Fig. 1, our discussions are equally valid for converting a T to an L wave. Eq. (4) suggests that *T*_T_ by the perfect TFPIs is only affected by the mechanical impedance ratio *ξ*. Similar behavior can be also observed in the classical FPIs sandwiched by a base medium of different material properties.

**Figure 2:**
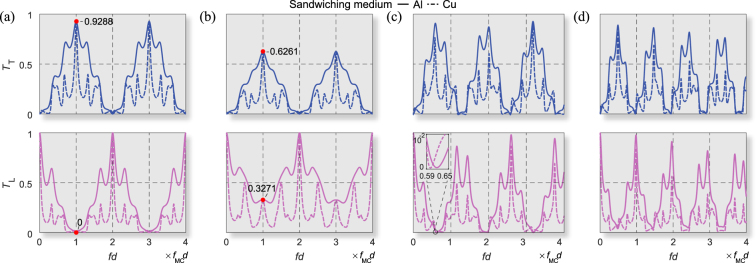
Transmission behaviors at different degrees of condition satisfaction of Eqs (1, 2) (*f*_MC_ *d*  = 2.45 kHz·m). (**a**) Simultaneous satisfaction of Eqs (1, 2), where *C*_11_ = *C*_66_ = 13 GPa and *C*_16_ = 5 GPa. (**b**) Satisfaction of Eq. (1) and violation of Eq. (2), where *C*_11_ = 16 GPa, *C*_66_ = 10 GPa and *C*_16_ = 4 GPa. (**c**) Violation of Eq. (1) and satisfaction of Eq. (2), where *C*_11_ = *C*_66_ = 13 GPa and *C*_16_ = 7 GPa. (**d**) No equations satisfied, where *C*_11_ = 13 GPa, *C*_66_ = 10 GPa and *C*_16_ = 7 GPa. In (**a**) and (**b**), Eq. (1) is satisfied for *n*_FS_ = 2 and *n*_SS_ = 3. The slabs are modeled as effective anisotropic mediums with a density *ρ* = 3000 kg/m^3^ sandwiched by aluminum (*ν*_Al_ = 0.33) or copper (*E*_Cu_ = 195 GPa, *ρ*_Cu_ = 9860 kg/m^3^, *ν*_Cu_ = 0.33).

Figure 2 shows how the *T*_T_ and *T*_L_ curves are altered if Eqs (1, 2) are not fully satisfied. Clearly, PMC cannot occur unless Eqs (1, 2) are simultaneously satisfied. Figure 2a also supports the finding from Eq. (4) that *T*_T_ is only affected by *ξ*, not related to material properties of the perfect TFPIs or other properties of the base medium. If only Eq. (1) is satisfied (see Fig. 2b), the transmission curves still exhibit repeated peaks similar to those in Fig. 2a. However, *T*_L_ is not zero at the PMC frequencies and *T*_T_ is much smaller than the corresponding values appearing in Fig. 2a. If only Eq. (2) is satisfied (see Fig. 2c), the transmission is no longer in an interference pattern, as *d* is not a multiple of the half-wavelengths. In this case, the material properties of adjacent isotropic medium significantly affect *T*_T_ and *T*_L_. At some frequencies, *T*_L_ is very close to, but not exactly zero. If the conditions described in Eqs (1, 2) are both violated (see Fig. 2d), no noticeable pattern is observed. While the earlier work^20^ indicated the possibility of the quasi-TFPR by considering only the phase difference between two coupled modes and some assumptions, the conditions (1) and (2) derived in this paper must be fulfilled exactly in order to obtain true Fabry-Perot interference pattern of mode conversion. The observations from Fig. 2 support that Eq. (1) is responsible for the interference phenomenon, whereas Eq. (2) is responsible for the maximum mode conversion and zero same-mode transmission at the mode-converting interference frequencies.

**Figure 3:**
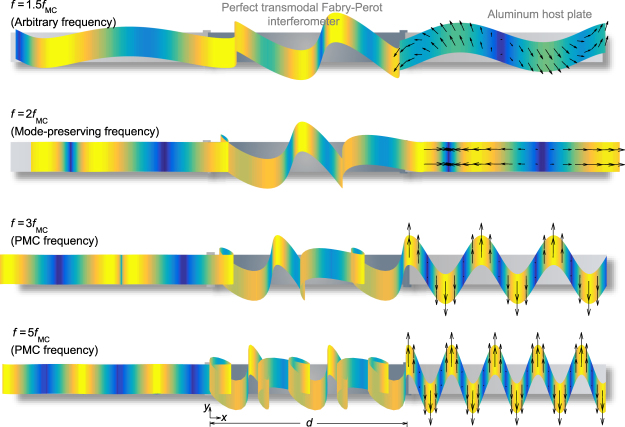
Snapshots of the transient displacement fields at various frequencies (*f*) for an L wave incidence using the interferometer depicted in Fig. 1. The parameters are the same as in Fig. 1, and the calculations were performed using the effective properties of the interferometer. The snapshots depict events at *t* = 29.25/*f* (see the Supplementary Movies S2–S4 for the displacement fields corresponding to *f* = 2*f*_MC_, 3*f*_MC_ and 5*f*_MC_ respectively).

To compare the mode conversion phenomenon at PMC frequencies (*f*_MC_, 3*f*_MC_, …) with the wave transmission phenomena at other frequencies, Fig. 3 presents the transient behaviors during wave transmission through the interferometer designed for the problem described in Fig. 1. The L-wave incidence is considered at various frequencies. The incident L wave leaves the interferometer as a pure L wave with 100% transmission at *f* = 2*f*_MC_. Although no apparent mode conversion occurs at *f* = 2*f*_MC_, L-T coupled skew motions occur inside the interferometer, unlike the classical single-mode Fabry-Perot interference.

**Figure 4:**
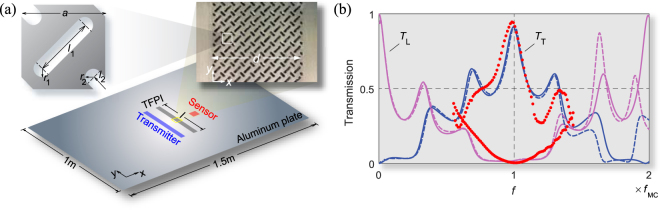
Experimental results. (**a**) Image of the anisotropic metamaterial slab used in the experiment (width *d* = 31.5 mm, length *l* = 400 mm) fabricated on a 1-mm-thick aluminum plate. The geometric parameters of the unit cell are *a* = 3.5 mm, *l*_1_ = 0.73*a*, *l*_2_ = 0.20*a*, and *r*_1_ = *r*_2_ = 0.1*a*. The lattice constant *a* is much smaller than the wavelength *λ*_SS_ (21.0 mm) at *f* *=* *f*_MC_. (**b**) *T*_T_ and *T*_L_ curves under an incident L wave around the PMC frequency, *f*_MC_ = 90.46 kHz. Solid lines: theoretical analysis; dashed lines: full-wave numerical simulation; dots: experimental results.

To verify the actual occurrence of PMC, we conducted experiments using a 1-mm-thick aluminum plate (Fig. 4a). Nine unit cells were fabricated in the *x* direction (i.e., the wave propagation direction; see the supplementary file) for experimental details). The unit cell of the fabricated interferometer in Fig. 4a was slightly modified from the unit cell shown in Fig. 1c to facilitate machining. The effective properties of the fabricated unit cell are *C*_11_ = 10.683 GPa, *C*_66_ = 11.921 GPa, *C*_16_ = 4.393 GPa, and *ρ* = 1920 kg/m^3^, which satisfy Eqs (2, 3) favorably. Satisfactory correlation is observed between the experimental results (*T*_T_ = 0.946 and *T*_L_ = 0.004 at *f* = *f*_MC_) and the theoretical/numerical predictions (*T*_T_ = 0.916 and *T*_L_ = 0.013 at *f* = *f*_MC_), as demonstrated in Fig. 4b. Because Eq. (2) is not fully satisfied, *T*_L_ does not reach zero.

## Discussion

To conclude, we derived the exact theory for the perfect TFPI, expressed it explicitly with two sets of equations and explained the mechanics occurring at the PMC frequencies. Although the pattern of peak mode-conversion transmissions in the TFPI is similar to that in the single-mode FPI, unique multimodal interactions must be considered in the TFPI. The phenomenon can be a promising strategy for freely transferring wave energy between dissimilar wave modes and thus the perfect interferometers can be a very useful device in industrial applications. Examples include its use in suppressing sound transmission through a panel, as acoustic waves in fluids (air) can transmit only longitudinal (or pressure) waves (see the supplementary information for simulation results). The TFPI can be also used for efficient generation or detection of difficult-to-generate and difficult-to-sense transverse waves for ultrasonic medical and industrial applications. The investigation into three-dimensional or non-collinear TFPIs may result in advanced and unexplored wave manipulation techniques and devices. Furthermore, this multimodal interference can be used in other physics disciplines that involve two or more wave modes or types.

## Methods and Materials

### Numerical simulations

For all the numerical simulations throughout the work, we utilized the commercial software COMSOL Multiphysics 5.2. In the transient simulation in Figs 1a and 3, the upper and lower boundaries are set to be Floquet-periodic to ensure the plane wave assumption, and the side boundaries are defined to be low-reflecting. Similar numerical model is used to obtain the full-wave transmission results in Figs 1b and 4b, except that the low-reflecting boundary conditions are replaced by perfectly matched layers (PMLs).

### Experimental set-up

The TFPI based on the unit cell shown in Fig. 4 was fabricated in a host aluminum (A6061-T6) plate of size 1500 mm × 1000 mm × 1 mm. Laser beam cutting was used to make voids in the unit cell. The interferometer has the dimension of 400 mm × 31.5 mm. It is made of 114 × 9 unit cells. Both the transmitting transducer (denoted by the transmitter in Fig. 4) and receiving transducer (denoted by the sensor in Fig. 4) used for the experiments operate by the principle of magnetostriction (the coupling phenomenon involving a magnetization process and dimension/shape change in ferromagnetic materials). Specifically, MPTs (Magnetostrictive Patch Transducers)^29^ are used, which consist of thin magnetostrictive patches, permanent magnets and electric coils.

In the frequency range of interest between 50 kHz and 130 kHz for which experiments were carried out, the lowest symmetric Lamb wave mode (S0 mode) and shear-horizontal guided wave mode (SH0 mode) simulate well the longitudinal mode and shear mode in bulk media. For the S0 wave mode excitation, a wide MPT employing a large magnetostrictive nickel patch of size 360 mm × 34 mm was used. At the sensing location where the wave exiting the TFPI would pass through, two different types of MPT sensors were placed to measure the S0 mode and SH0 mode, respectively. The dimensions of the magnetostrictive nickel patches (e.g. nickel patches) used for the S0 and SH0 mode sensing are 30 mm × 36 mm and 38 mm × 36 mm, respectively. Shear couplant (Sonotech SHEAR GEL® MAGNAFLUX) or a double-sided bonding tape (3MTM) was used to couple the patch with the host plate. For stable transduction, some care must be taken to ensure uniform adhesion of the patch to the host plate.

To generate the S0 mode wave to be incident on the TFPI, 4 to 8 cycles of a sine wave were generated by a function generator. The main reason to use the truncated sine wave over a Gaussian-modulated sine wave having less side lobes in the frequency domain is that the former has a larger amplitude and a narrower bandwidth near the target frequency that the latter. Considering that limit on the allowed input power to the transducer, the truncated sine wave is therefore favorable for the present experiment. To calculate the normalized longitudinal transmission power, a reference signal of the S0 mode was obtained before the incident wave entered the interferometer. On the other hand, an indirect calibration method (See ref.^20^ for details) was used to calculate the normalized shear transmission power. This indirect calibration is inevitable because the SH0 mode wave can be measured only after the incident S0 mode wave passes through the TFPI. For the calibration, the frequency characteristics of the S0 mode actuators and the SH0 mode sensors were determined by additional experiments. The indirectly obtained shear transmittances were then fitted to the simulation results using the least square fitting. This fitting process is needed because one cannot quantitatively estimate how much the S0-mode signal (the input voltage of the actuator) can be converted through the TFPI to the SH0-mode output (the output voltage of the sensor). Therefore, we first compensated the frequency characteristics of the used transducers characteristics and then used the least square method to fit the indirectly-calibrated, measured shear mode transmittance to the numerically-obtained, normalized shear mode transmittance.

## Electronic supplementary material

Supplementary_file

normalized_transient_displacement_f=1fMC

normalized_transient_displacement_f=2fMC

normalized_transient_displacement_f=3fMC

normalized_transient_displacement_f=5fMC
