# Supplementary material for: Theory for Perfect Transmodal Fabry-Perot Interferometer
Source: Sci Rep. 2018 Jan 8;8:69. doi: 10.1038/s41598-017-18408-5 (PMC5758750; doi:10.1038/s41598-017-18408-5)
Supplement: Supplementary file 1 — Supplementary_file [file 41598_2017_18408_MOESM1_ESM.pdf]

Supplementary file

Theory for Perfect Transmodal Fabry-Perot Interferometer

Xiongwei Yang [1], Joshua M. Kweun [2], and Yoon Young Kim [1,2,3*].

[1]BK21 Plus Transformative Program for Creative Mechanical & Aerospace Engineers, Seoul National University, 1 Gwanak-ro, Gwanak-gu, Seoul 08826, South Korea.

[2]School of Mechanical and Aerospace Engineering, Seoul National University, 1 Gwanak-ro, Gwanak-gu, Seoul 08826, South Korea.

[3]Institute of Advanced Machines and Design, Seoul National University, 1 Gwanak-ro, Gwanak-gu, Seoul 08826, South Korea.

[*]Correspondence to: yykim@snu.ac.kr.

1. Theoretical analysis

1.1 Fundamental equations

Consider a mechanical wave propagating in a two-dimensional x-y plane through an anisotropic elastic slab of width d that is sandwiched between layers of another base medium. In the subsequent analysis, we will consider a longitudinal wave propagating along the x direction, which is normally incident from the base medium to the anisotropic slab. Before we derive the exact conditions for multimodal interference for Perfect Mode Conversion (PMC), the fundamental equations will be briefly explained.

Because waves propagating along the x axis will be considered, the stiffness coefficients needed for the analysis are C_{11}, C_{66}, and C_{16} for the slab medium. The stiffness terms with subscripts (11) and (66) denote the longitudinal stiffness along the x axis and the shear stiffness, respectively while the term with (16), the longitudinal-shear coupling term. If the base medium is isotropic, it will be characterized by Young's modulus (E_0) and Poisson's ratio (ν_0). If it is an anisotropic medium, it will be characterized by c_{11}, c_{66}, and c_{16}, but c_{16}=0 is assumed because purely longitudinal (transverse) wave incidence from the base medium to the sandwiched slab is considered. The symbols ρ_0 and ρ will be used to denote the densities of the base material and slab material, respectively. Because the actual anisotropic slab will be made of a metamaterial, the stiffness C_{ij} can be understood as effective stiffness.

For waves propagating along the x direction (see Figure. 1a of the main text), the x- and y-directional displacements u_x and u_y can be assumed to vary as $e^{j(kx-\omega t)}$ where k, ω and t are the x-directional wavenumber, angular frequency and time. The Christoffel equation [S1] can be written as

$$\begin{bmatrix} C_{11}k^2-\rho\omega^2 & C_{16}k^2 \\ C_{16}k^2 & C_{66}k^2-\rho\omega^2 \end{bmatrix}\begin{Bmatrix} u_x \\ u_y \end{Bmatrix}=0, \quad \text{(S1)}$$

Non-trivial solutions for Eq. (S1) can be found if $k=\pm\alpha$ or $k=\pm\beta$ where

$$\alpha=\sqrt{\frac{\rho\omega^2\left(C_{11}+C_{66}\right)-\rho\omega^2\sqrt{\left(C_{11}-C_{66}\right)^2+4C_{16}^2}}{2\left(C_{11}C_{66}-C_{16}^2\right)}},$$
$$\beta=\sqrt{\frac{\rho\omega^2\left(C_{11}+C_{66}\right)+\rho\omega^2\sqrt{\left(C_{11}-C_{66}\right)^2+4C_{16}^2}}{2\left(C_{11}C_{66}-C_{16}^2\right)}}. \quad \text{(S2)}$$

Therefore, the general solution in the anisotropic slab can be expressed as

$$u_x=\left(AP_x\,\mathrm{e}^{j\alpha x}+BP_x\,\mathrm{e}^{-j\alpha x}+CQ_x\mathrm{e}^{j\beta x}+DQ_x\mathrm{e}^{-j\beta x}\right)\mathrm{e}^{-j\omega t},$$
$$u_y=\left(AP_y\,\mathrm{e}^{j\alpha x}+BP_y\,\mathrm{e}^{-j\alpha x}+CQ_y\mathrm{e}^{j\beta x}+DQ_y\mathrm{e}^{-j\beta x}\right)\mathrm{e}^{-j\omega t}. \quad \text{(S3)}$$

The symbols A and B denote unknown amplitudes corresponding to $k=+\alpha$ and $k=-\alpha$, respectively. Likewise, C and D are unknown amplitudes corresponding to $k=+\beta$ and $k=-\beta$, respectively.

From Eq. (S3), the displacement components corresponding to the α and β waves can be expressed as

$$u_x^{\alpha}=\left(A\mathrm{e}^{j\alpha x}+B\mathrm{e}^{-j\alpha x}\right)P_x\,\mathrm{e}^{-j\omega t},u_y^{\alpha}=\left(A\mathrm{e}^{j\alpha x}+B\mathrm{e}^{-j\alpha x}\right)P_y\,\mathrm{e}^{-j\omega t},$$
$$u_x^{\beta}=\left(C\mathrm{e}^{j\beta x}+D\mathrm{e}^{-j\beta x}\right)Q_x\,\mathrm{e}^{-j\omega t},u_y^{\beta}=\left(C\mathrm{e}^{j\beta x}+D\mathrm{e}^{-j\beta x}\right)Q_y\,\mathrm{e}^{-j\omega t}, \quad \text{(S4)}$$

where P_x, P_y, Q_x, and Q_y represent the polarization vector components. They are found to be

$$P_x=\frac{X_\alpha}{\sqrt{1+\left|X_\alpha\right|^2}},P_y=\frac{1}{\sqrt{1+\left|X_\alpha\right|^2}},Q_x=\frac{X_\beta}{\sqrt{1+\left|X_\beta\right|^2}},Q_y=\frac{1}{\sqrt{1+\left|X_\beta\right|^2}}, \quad \text{(S5)}$$

where

$$X_k=-\frac{C_{16}k^2}{C_{11}k_x^2-\rho\omega^2}=-\frac{C_{66}k^2-\rho\omega^2}{C_{16}k_x^2}\left(k=\pm\alpha,\pm\beta\right). \quad \text{(S6)}$$

The symbol X_k represents the amplitude ratio of u_x and u_y. Due to the orthogonality of the wave mode, the relation that $P_xQ_x+P_yQ_y=0$ always holds .

One can express the velocity (v_x and v_y) and stress (σ_{xx} and σ_{xy}) as

$$\begin{Bmatrix} v_x \\ v_y \\ \sigma_{xx} \\ \sigma_{xy} \end{Bmatrix}=\mathbf{MN}\begin{Bmatrix} A \\ B \\ C \\ D \end{Bmatrix}. \quad \text{(S7)}$$

Please see Appendix for the elements of the **M** and **N** matrices. If C_{ij} and ρ are replaced by c_{ij} and ρ_0 in the equations above, the corresponding results are applicable for the base medium. In this case, the x-directional wave numbers will be denoted α_0 and β_0.

1.2 Transmissions and reflections

To obtain the scattering (S) parameters, we first write the relationship between the velocity and stress fields on the left and the right boundaries of the anisotropic slab (see Figure.1a of the main text),

$$\begin{Bmatrix} v_x \\ v_y \\ \sigma_{xx} \\ \sigma_{xy} \end{Bmatrix}_{x=d^-} = \mathbf{M}\mathbf{N}_{x=d}\mathbf{M}^{-1} \begin{Bmatrix} v_x \\ v_y \\ \sigma_{xx} \\ \sigma_{xy} \end{Bmatrix}_{x=0^+} \triangleq \mathbf{T}_{x=d} \begin{Bmatrix} v_x \\ v_y \\ \sigma_{xx} \\ \sigma_{xy} \end{Bmatrix}_{x=0^+} . \tag{S8}$$

By imposing the continuities between the field variables of the base medium and those of the anisotropic slab at *x=0* and *x=d*, we can obtain the **S** matrix as

$$\mathbf{S} = \mathbf{M}_0^{-1}\mathbf{T}_{x=d}\mathbf{M}_0, \tag{S9}$$

where $\mathbf{M}_0$ is the **M** matrix for the base medium. The **S** matrix represents the relationship between the displacement amplitudes of the adjacent base medium at x=0^- and those at x=d^+.

Once the components (S_{ij}) of the **S** matrix are determined, the reflection and transmission coefficients for the case of longitudinal wave incidence can be expressed as:

$$\begin{aligned} RLL &= \frac{S_{24}S_{41} - S_{44}S_{21}}{S_{22}S_{44} - S_{42}S_{24}}, \\ RLT &= \frac{S_{42}S_{21} - S_{22}S_{41}}{S_{22}S_{44} - S_{42}S_{24}}, \\ TLL &= S_{11} + S_{12}RLL + S_{14}RLT, \\ TLT &= S_{31} + S_{32}RLL + S_{34}RLT. \end{aligned} \tag{S10}$$

In Eq. (S10), *RLL* and *RLT* denote the reflection coefficients for the reflected L and T waves, respectively, and *TLL* and *TLT,* the transmission coefficients for transmitted L and T waves, respectively. The explicit expressions for the elements of the **T** and **S** matrices are given in Appendix.

The mode conversion ratio, i.e. the L-to-T transmission power ratio (T_{T}) represents the ratio of the transmitted S-wave power intensity (P^{TLT}) to the incident L-wave power intensity (P^{I}). Therefore, it can be expressed as

$$T_{\mathrm{T}} = \frac{P^{\mathrm{TLT}}}{P^{\mathrm{I}}} = \frac{\mathrm{real}\left(\sigma_{xy}^{\mathrm{TLT}} \times \mathrm{conj}\left(v_y^{\mathrm{TLT}}\right)\right)|_{x=d^+}}{\mathrm{real}\left(\sigma_{xx}^{\mathrm{I}} \times \mathrm{conj}\left(v_x^{\mathrm{I}}\right)\right)|_{x=0^-}} = \xi\left|TLT\right|^2, \tag{S11}$$

where

$$\xi \triangleq \frac{\bar{\beta}_0}{\bar{\alpha}_0},\ \bar{\alpha}_0 \triangleq \alpha_0 c_{11},\ \bar{\beta}_0 \triangleq \beta_0 c_{66}. \tag{S12}$$

Likewise, the same-mode L-to-L transmission power ratio (T_{L}) representing the ratio of the transmitted T-wave power intensity (P^{TLL}) to the incident L-wave power intensity (P^{I}) can be obtained as

$$T_{\mathrm{L}} = \frac{P^{\mathrm{TLL}}}{P^{\mathrm{I}}} = \frac{\mathrm{real}\left(\sigma_{xx}^{\mathrm{TLL}} \times \mathrm{conj}\left(v_x^{\mathrm{TLL}}\right)\right)|_{x=d^+}}{\mathrm{real}\left(\sigma_{xx}^{\mathrm{I}} \times \mathrm{conj}\left(v_x^{\mathrm{I}}\right)\right)|_{x=0^-}} = \left|TLL\right|^2. \tag{S13}$$

Similarly, the reflections of the L wave and T wave can be written as

$$R_{\mathrm{L}} = \left|RLL\right|^2, R_{\mathrm{T}} = \xi\left|RLT\right|^2. \tag{S14}$$

1.3 Perfect Mode Conversion with Full Transmission

In this section, we will derive the condition for perfect mode conversion with full transmission (i.e., with T_T=100%). As mentioned earlier, we will mainly consider the L-wave incidence but the obtained result is equally valid for the S-wave incidence. For perfect mode conversion, one mode can be perfectly converted only to the other mode, i.e. T_L=0 at PMC (Perfect Mode Conversion) frequencies. T_T can reach 100% if the mechanical impedances of the longitudinal and transverse waves in the base medium are the same. We begin with this special case before discussing more general cases of perfect mode conversion.

As we are interested in the incidence of a pure L wave from a base medium, no coupling between longitudinal and transverse stiffness is assumed, i.e., c_{16}=0. In this section, the base medium is assumed to be anisotropic.

Because T_T given in Eq. (S11) is very complicated, to find the exact condition for the perfect mode conversion, we use the following equation for sake of simplification based on the observation [20] that the L-to-L transmission could nearly vanish at some frequencies

$$C_{11} = C_{66}. \tag{S15}$$

The **S** matrix (see Appendix) can then reduce to

$$\mathbf{S} = \begin{bmatrix} V_1 - \frac{\mathrm{j}\omega V_5}{2\bar{\alpha}_0} - \frac{\mathrm{j}\bar{\alpha}_0 V_3}{2\omega} & -\frac{\mathrm{j}\omega V_5}{2\bar{\alpha}_0} + \frac{\mathrm{j}\bar{\alpha}_0 V_3}{2\omega} & (1+\xi)\frac{V_2}{2} - \frac{\mathrm{j}\omega V_6}{2\bar{\alpha}_0} - \frac{\mathrm{j}\bar{\beta}_0 V_4}{2\omega} & (1-\xi)\frac{V_2}{2} - \frac{\mathrm{j}\omega V_6}{2\bar{\alpha}_0} + \frac{\mathrm{j}\bar{\beta}_0 V_4}{2\omega} \\ \frac{\mathrm{j}\omega V_5}{2\bar{\alpha}_0} - \frac{\mathrm{j}\bar{\alpha}_0 V_3}{2\omega} & V_1 + \frac{\mathrm{j}\omega V_5}{2\bar{\alpha}_0} + \frac{\mathrm{j}\bar{\alpha}_0 V_3}{2\omega} & (1-\xi)\frac{V_2}{2} + \frac{\mathrm{j}\omega V_6}{2\bar{\alpha}_0} - \frac{\mathrm{j}\bar{\beta}_0 V_4}{2\omega} & (1+\xi)\frac{V_2}{2} + \frac{\mathrm{j}\omega V_6}{2\bar{\alpha}_0} + \frac{\mathrm{j}\bar{\beta}_0 V_4}{2\omega} \\ \left(1+\frac{1}{\xi}\right)\frac{V_2}{2} - \frac{\mathrm{j}\omega V_6}{2\bar{\beta}_0} - \frac{\mathrm{j}\bar{\alpha}_0 V_4}{2\omega} & \left(1-\frac{1}{\xi}\right)\frac{V_2}{2} - \frac{\mathrm{j}\omega V_6}{2\bar{\beta}_0} + \frac{\mathrm{j}\bar{\alpha}_0 V_4}{2\omega} & V_1 - \frac{\mathrm{j}\omega V_5}{2\bar{\beta}_0} - \frac{\mathrm{j}\bar{\beta}_0 V_3}{2\omega} & -\frac{\mathrm{j}\omega V_5}{2\bar{\beta}_0} + \frac{\mathrm{j}\bar{\beta}_0 V_3}{2\omega} \\ \left(1-\frac{1}{\xi}\right)\frac{V_2}{2} + \frac{\mathrm{j}\omega V_6}{2\bar{\beta}_0} - \frac{\mathrm{j}\bar{\alpha}_0 V_4}{2\omega} & \left(1+\frac{1}{\xi}\right)\frac{V_2}{2} + \frac{\omega V_6}{2\bar{\beta}_0} + \frac{\bar{\alpha}_0 V_4}{2\omega} & \frac{\mathrm{j}\omega V_5}{2\bar{\beta}_0} - \frac{\mathrm{j}\bar{\beta}_0 V_3}{2\omega} & V_1 + \frac{\mathrm{j}\omega V_5}{2\bar{\beta}_0} + \frac{\mathrm{j}\bar{\beta}_0 V_3}{2\omega} \end{bmatrix} \tag{S16}$$

where

$$\begin{aligned} &V_1 \triangleq \frac{1}{2}\left[\cos(\alpha d) + \cos(\beta d)\right], \qquad V_2 \triangleq \frac{1}{2}\left[\cos(\alpha d) - \cos(\beta d)\right], \\ &V_3 \triangleq \frac{-1}{2\rho\omega}\left[\alpha\sin(\alpha d) + \beta\sin(\beta d)\right], \; V_4 \triangleq \frac{-1}{2\rho\omega}\left[\alpha\sin(\alpha d) - \beta\sin(\beta d)\right], \\ &V_5 \triangleq \frac{-\rho\omega}{2}\left[\frac{\sin(\alpha d)}{\alpha} + \frac{\sin(\beta d)}{\beta}\right], \qquad V_6 \triangleq \frac{-\rho\omega}{2}\left[\frac{\sin(\alpha d)}{\alpha} - \frac{\sin(\beta d)}{\beta}\right]. \end{aligned} \tag{S17}$$

As the components S_{ij} in Eq. (S16) still appear to be complicated, we consider the case of a special anisotropic base material satisfying

$$c_{11} = c_{66}. \tag{S18}$$

This condition is equivalent to the condition that the mechanical impedance of the longitudinal wave is equal to that of the transverse wave. With Eq. (S18) (and c_{16}=0), one can have ξ=1. In this case, Eq. (S16) can be simplified to

$$\mathbf{S}=\begin{bmatrix} V_1-\frac{\mathrm{j}\omega V_5}{2\bar{\alpha}_0}-\frac{\mathrm{j}\bar{\alpha}_0V_3}{2\omega} & -\frac{\mathrm{j}\omega V_5}{2\bar{\alpha}_0}+\frac{\mathrm{j}\bar{\alpha}_0V_3}{2\omega} & V_2-\frac{\mathrm{j}\omega V_6}{2\bar{\alpha}_0}-\frac{\mathrm{j}\bar{\alpha}_0V_4}{2\omega} & -\frac{\mathrm{j}\omega V_6}{2\bar{\alpha}_0}+\frac{\mathrm{j}\bar{\alpha}_0V_4}{2\omega} \\ -S_{12} & \langle S_{11}\rangle & -S_{14} & \langle S_{13}\rangle \\ S_{13} & S_{14} & S_{11} & S_{12} \\ -S_{14} & \langle S_{13}\rangle & -S_{12} & \langle S_{11}\rangle \end{bmatrix}, \quad \text{(S19)}$$

where the symbol <> denotes the complex conjugate of the term inside. Clearly, T_T can reach 100%, only if RLL=0, RLT=0, TLL=0 and TLT=±1. These conditions can be converted to the following equations

$$\begin{cases} S_{24}S_{41}-S_{44}S_{21}=0 \\ S_{42}S_{21}-S_{22}S_{41}=0 \\ S_{11}=0 \\ S_{31}=\pm1 \end{cases} \Rightarrow \begin{cases} -\langle S_{13}\rangle S_{14}-\langle S_{11}\rangle S_{21}=0 \\ -\langle S_{13}\rangle S_{12}+\langle S_{11}\rangle S_{14}=0 \\ S_{11}=0 \\ S_{31}=\pm1 \end{cases} \Rightarrow \begin{cases} -\langle S_{13}\rangle S_{14}=0 \\ -\langle S_{13}\rangle S_{12}=0 \\ S_{11}=0 \\ S_{13}=\pm1 \end{cases} \Rightarrow \begin{cases} S_{14}=0 \\ S_{12}=0 \\ S_{11}=0 \\ S_{13}=\pm1 \end{cases}$$

$$\Rightarrow \begin{cases} \frac{\mathrm{j}\omega}{\bar{\alpha}_0}V_6-\frac{\mathrm{j}\bar{\alpha}_0}{\omega}V_4=0 \\ \frac{\mathrm{j}\omega}{\bar{\alpha}_0}V_5-\frac{\mathrm{j}\bar{\alpha}_0}{\omega}V_3=0 \\ V_1-\frac{\mathrm{j}\omega}{2\bar{\alpha}_0}V_5-\frac{\mathrm{j}\bar{\alpha}_0}{2\omega}V_3=0 \\ V_2-\frac{\mathrm{j}\omega}{2\bar{\alpha}_0}V_6-\frac{\mathrm{j}\bar{\alpha}_0}{2\omega}V_4=\pm1 \end{cases} \Rightarrow \begin{cases} \frac{\mathrm{j}\omega}{\bar{\alpha}_0}V_6-\frac{\mathrm{j}\bar{\alpha}_0}{\omega}V_4=0 \\ \frac{\mathrm{j}\omega}{\bar{\alpha}_0}V_5-\frac{\mathrm{j}\bar{\alpha}_0}{\omega}V_3=0 \\ \frac{\mathrm{j}\omega}{\bar{\alpha}_0}V_5+\frac{\mathrm{j}\bar{\alpha}_0}{\omega}V_3=0 \\ V_1=0 \\ V_2-\frac{\mathrm{j}\omega}{2\bar{\alpha}_0}V_6-\frac{\mathrm{j}\bar{\alpha}_0}{2\omega}V_4=\pm1 \end{cases}. \quad \text{(S20)}$$

Note that we used Eq. (S10) to obtain (S20). Because all V_i (i=1,2,3,…,6) are real, Eq. (S20) requires that

$$V_1=0;\ V_2=\pm1;\ V_3=V_4=V_5=V_6=0. \quad \text{(S21)}$$

If the following conditions are met, Eq. (S21) can be identically satisfied:

$$\cos(\alpha d)=\pm1,\ \cos(\beta d)=\mp1. \quad \text{(S22)}$$

To extract more useful information from (S22), the explicit expressions for α and β are written

$$\begin{aligned} &\alpha d=N_{\mathrm{FS}}\pi, \\ &\beta d=N_{\mathrm{SS}}\pi, \\ &(N_{\mathrm{FS}}<N_{\mathrm{SS}}:\ \text{Integers with } N_{\mathrm{FS}}+N_{\mathrm{SS}}=\text{odd}). \end{aligned} \quad \text{(S23)}$$

Because $\alpha\leq\beta$, N_{SS} must be larger than N_{FS}. If Eq. (S23) is expressed in terms of the wavelengths λ_{FS} and λ_{SS}, we have

$$\begin{aligned} d&=\frac{N_{\mathrm{FS}}}{2}\lambda_{\mathrm{FS}}, \\ d&=\frac{N_{\mathrm{SS}}}{2}\lambda_{\mathrm{SS}}. \end{aligned} \quad \text{(S24)}$$

Eq. (S24) states that perfect and full (100%) conversion from the L wave mode to the T wave mode is possible if the slab width d is a multiple of half the wavelength λ_{FS} and a multiple of

half the wavelength of λ_{SS} simultaneously.

If the coprime integers of N_{FS} and N_{SS} are denoted by n_{FS} and n_{SS} with the corresponding fundamental PMC frequency f_{MC} Eq. (S23) can be written as

$$\begin{aligned} &\alpha d = N_{FS}\pi = m n_{FS}\pi, \\ &\beta d = N_{SS}\pi = m n_{SS}\pi, \\ &n_{FS} + n_{SS} = \text{odd} \end{aligned} \tag{S25}$$

where m=1, 3, 5, … and coprime integers n_{FS} and n_{SS} satisfy $n_{FS} < n_{SS}$. The conditions for PMC , i.e. Eq. (S24), can be put in more convenient form as

$$\begin{aligned} &d = \frac{N_{FS}}{2}\lambda_{FS} = \frac{m}{2} n_{FS}\lambda_{FS}, \\ &d = \frac{N_{SS}}{2}\lambda_{SS} = \frac{m}{2} n_{SS}\lambda_{SS}, \\ &n_{FS} + n_{SS} = \text{odd } (n_{FS} < n_{SS} : \text{coprime integers}) \end{aligned} \tag{S26}$$

with m=1, 3, 5, …

We can find that for perfect and full (100%) mode conversion, the slab should serve as a multimodal interferometer. For the condition given by Eq. (S26) to hold**,** the stiffness coefficients (C_{ij}) of the sandwiched interferometer must satisfy the following relations

$$\begin{aligned} &\Gamma \triangleq \frac{C_{11} + C_{66}}{2} = \frac{C_{MC}}{2}\left(\frac{1}{n_{FS}^2} + \frac{1}{n_{SS}^2}\right), \\ &\Pi \triangleq \sqrt{C_{11}C_{66} - C_{16}^2} = \frac{C_{MC}}{n_{FS}n_{SS}}, \end{aligned} \tag{S27}$$

where

$$C_{MC} \triangleq 4\rho f_T^2 d^2. \tag{S28}$$

To obtain Eq. (S27), α and β in Eq. (S25) were expressed as (with m=1)

$$\alpha = \frac{n_{FS}\pi}{d}, \beta = \frac{n_{SS}\pi}{d}. \tag{S29}$$

Solving Eq. (S2) with Eq. (S29), one can obtain Eq. (S27).

The analysis above show that the conditions for *perfect* and *full* (100%) mode-conversion transmission (T_T=1 and T_L=0) are obtained as Eqs. (S15) and (S26) that apply to the interferometer slab and Eq. (S18) that applies to the base medium. FIG. S1 shows the transmission curves exhibiting interference behavior when perfect and full mode conversion occurs. At the PMC frequencies f=f_{MC}, 3f_{MC}, ⋯, T_T=100% and T_L = 0.

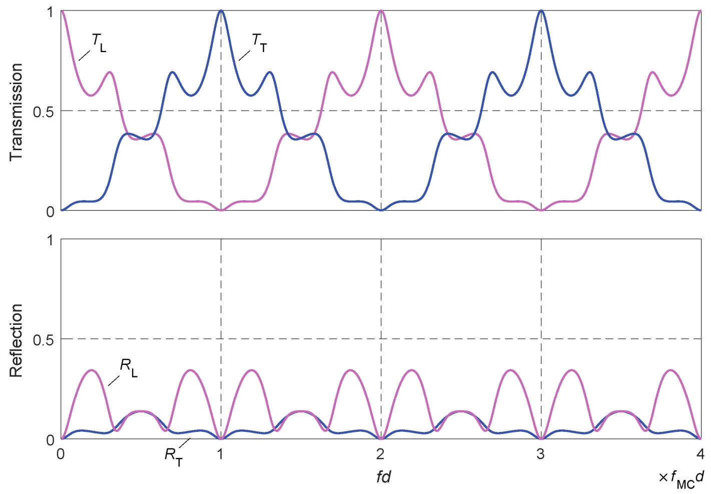

FIG. S1 Perfect and full mode conversion yielding 100% L-to-T mode conversion at PMC frequencies f=f_{MC}, 3f_{MC},⋯.The TFPI satisfies Eq. (S15) and Eq. (S26) (or Eq. (S27)) with n_{FS} = 2, n_{SS} = 3, and $f_{MC}d$ =2.45 kHz·m. C_{11}=C_{66}=13GPa, C_{16}=5GPa, ρ=3000kg/m^3. The base medium fulfills Eq. (S18); for the present simulation, c_{11}=c_{66}=60GPa, c_{16}=0, and ρ_0=3000kg/m^3 are used.

1.4 Theory of perfect mode conversion

The conditions for full and perfect mode conversion derived in the previous section require a special anisotropic base medium satisfying c_{11}=c_{66} and c_{16}=0. This base material requirement is very restrictive. Now, we consider general isotropic base media and look for the conditions for PMC in which 100% transmodal transmission is relaxed. Therefore, we will remove the condition given by Eq. (S18) imposed on the base medium and consider the two remaining conditions given by Eqs. (S26) and (S15) imposed on the TFPI modeled as an anisotropic slab.

First, let us consider the condition (S26), equivalently, (S22). Without the loss of generality, only the following equations can be considered

$$\cos(\alpha d)=1,\ \cos(\beta d)=-1. \tag{S30}$$

The frequencies that satisfy Eq. (S30) will be denoted by f_{MC}, 3f_{MC}, …, as before. When Eq. (S30) is substituted into the **S** matrix given in Appendix, we have

$$\mathbf{S}=\begin{bmatrix} W_1 & 0 & (1+\xi)\frac{W_2}{2} & (1-\xi)\frac{W_2}{2} \\ 0 & W_1 & (1-\xi)\frac{W_2}{2} & (1+\xi)\frac{W_2}{2} \\ \left(1+\frac{1}{\xi}\right)\frac{W_2}{2} & \left(1-\frac{1}{\xi}\right)\frac{W_2}{2} & -W_1 & 0 \\ \left(1-\frac{1}{\xi}\right)\frac{W_2}{2} & \left(1+\frac{1}{\xi}\right)\frac{W_2}{2} & 0 & -W_1 \end{bmatrix}, \tag{S31}$$

where

$$W_1 = \frac{1}{2}\left(P_x Q_y + P_y Q_x\right), W_2 = 2P_y Q_y. \tag{S32}$$

If Eq.(S31) is used, *TLT* and *TLL* at the PMC frequencies f=f_{MC}, $3f_{MC}$, … become

$$TLT\left(f_{\text{MC}}, 3f_{\text{MC}}, \cdots\right) = \left[1 - \frac{1}{\frac{4\xi}{(1-\xi)^2}\frac{W_1^2}{W_2^2} + \left(\frac{1+\xi}{1-\xi}\right)^2}\right]\frac{\xi+1}{2\xi}W_2, \tag{S33}$$

$$TLL\left(f_{\text{MC}}, 3f_{\text{MC}}, \cdots\right) = \left[1 - \frac{1}{\frac{4\xi}{(\xi-1)^2}\frac{W_1^2}{W_2^2} + \left(\frac{\xi+1}{\xi-1}\right)^2 W_2^2}\right]W_1. \tag{S34}$$

Let us now consider the consequence of Eq. (S15). (Recall that Eq. (S26) is assumed to hold.) In this case, the components of the polarization vector become

$$\begin{aligned} &P_x = \frac{\sqrt{2}}{2}, P_y = \frac{\sqrt{2}}{2}, Q_x = -\frac{\sqrt{2}}{2}, Q_y = \frac{\sqrt{2}}{2}, \text{when } C_{16} > 0; \\ &P_x = -\frac{\sqrt{2}}{2}, P_y = \frac{\sqrt{2}}{2}, Q_x = \frac{\sqrt{2}}{2}, Q_y = \frac{\sqrt{2}}{2}, \text{when } C_{16} < 0; \end{aligned} \tag{S35}$$

and

$$W_1 = 0, W_2 = 1. \tag{S36}$$

Substituting Eq. (S36) into Eq. (S33) and Eq. (S34) yields

$$TLT\left(f_{\text{MC}}, 3f_{\text{MC}}, \cdots\right) = \frac{\xi+1}{2\xi}\left[1 - \left(\frac{1-\xi}{1+\xi}\right)^2\right], \tag{S37}$$

$$TLL\left(f_{\text{MC}}, 3f_{\text{MC}}, \cdots\right) = 0. \tag{S38}$$

For an isotropic base medium (E_0, v_0), the parameter ξ appearing in Eq. (S37) becomes

$$\xi = \frac{\beta_0 c_{66}}{\alpha_0 c_{11}} = \sqrt{\frac{1-2\nu_0}{2\left(1-\nu_0\right)}} \text{ (plane-strain case)}, \tag{S39}$$

$$\xi = \sqrt{\frac{1-\nu_0}{2}} \text{ (plane-stress case)}. \tag{S40}$$

To obtain Eq. (S39), we used the relationship,

$$c_{11} = \frac{E_0\left(1-\nu_0\right)}{\left(1+\nu_0\right)\left(1-2\nu_0\right)}, c_{66} = \frac{E_0}{2\left(1+\nu_0\right)}. \tag{S41}$$

Once Eq. (S39) for the plane-strain case is obtained, it is straightforward to convert it to the result for the plane-stress case by replacing v_0 with $v_0/(v_0+1)$.

At this point, it is worth summarizing the findings for PMC. For PMC to occur, the following conditions must be satisfied

$$d = \frac{N_{\mathrm{FS}}}{2}\lambda_{\mathrm{FS}} = \frac{m}{2} n_{\mathrm{FS}}\lambda_{\mathrm{FS}},$$
$$d = \frac{N_{\mathrm{SS}}}{2}\lambda_{\mathrm{SS}} = \frac{m}{2} n_{\mathrm{SS}}\lambda_{\mathrm{SS}}, \tag{S42}$$
$$n_{\mathrm{FS}} + n_{\mathrm{SS}} = \text{odd}\ ({}_{\mathrm{FS}} < n_{\mathrm{SS}}\text{: coprime integers})$$

with m=1,3,5…. and

$$C_{11} = C_{66}. \tag{S43}$$

Then, the transmission coefficients and power transmission ratios at the PMC frequencies (f_{MC}, $3f_{\mathrm{MC}}$) become

$$TLT\left(f_{\mathrm{MC}}, 3f_{\mathrm{MC}}, \cdots\right) = \frac{\xi+1}{2\xi}\left[1-\left(\frac{1-\xi}{1+\xi}\right)^{2}\right], TLL\left(f_{\mathrm{MC}}, 3f_{\mathrm{MC}}, \cdots\right) = 0. \tag{S44}$$

$$T_{\mathrm{T}}\left(f_{\mathrm{MC}}, 3f_{\mathrm{MC}}, \cdots\right) = \xi\left|TLT\right|^{2}, T_{\mathrm{L}}\left(f_{\mathrm{MC}}, 3f_{\mathrm{MC}}, \cdots\right) = 0; \tag{S45}$$

It is obvious that T_{T} are maximized at the PMC frequencies and T_{L}=0. However, T_{T} cannot be 100% in general because it depends on Poisson's ratio v_0 of the base medium. FIG. S2 shows the effect of v_0 on T_{T} and T_{L} at the PMC frequencies. It can be found that for the plane-stress case, T_{T} at the PMC frequencies approaches 100% as v_0 approaches -1. It is apparent that T_{L} always vanishes at the PMC frequencies in either case, leading to perfect L-to-T mode conversion.

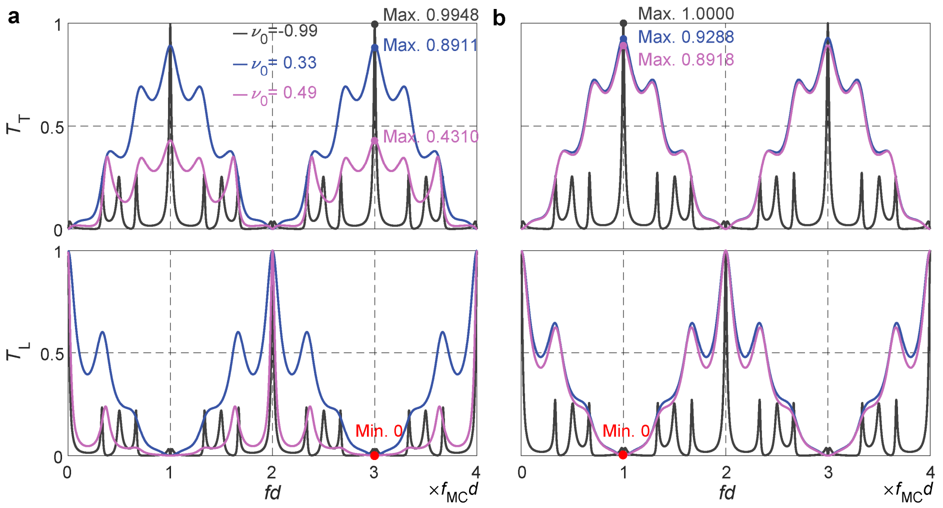

FIG. S2 Transmission at the PMC frequencies with different υ_0. (A) Plane-strain case. (B) Plane-stress case. The effective properties of the TFPI satisfy Eq. (S42) and Eq. (S43) with n_{FS}=2, n_{SS}=3, and f_{MC}d=2.45 kHz·m. C_{11}=C_{66}=13GPa, C_{16}=5GPa, ρ=3000kg/m^3. The material properties of the background medium are assumed to have E_0=60Gpa and ρ_0=3000kg/m^3.

1.5 Remarks on displacement field inside the interferometer at frequency $2f_{MC}$

It has been shown in Figure. 1 of the main text that at the frequencies, $2f_{MC}$, $4f_{MC}$,… R_L=R_T=T_T=0 and T_L=1. This means that at these frequencies, the incident wave mode is preserved after it passes through the TFPI. To illustrate the mode-preserving mechanism through the TFPI, we calculate the distributions of u_x and u_y displacement components inside the TFPI in FIG. S3. It shows that the u_x components of the FS and SS modes can interfere constructively while their u_y components interfere destructively. This interference behavior at f=$2f_{MC}$, $4f_{MC}$, … differs from that observed at f=f_{MC}, $3f_{MC}$,…, in which the u_y components interfere constructively while the u_x components interfere destructively (see Figure 1e in the main text).

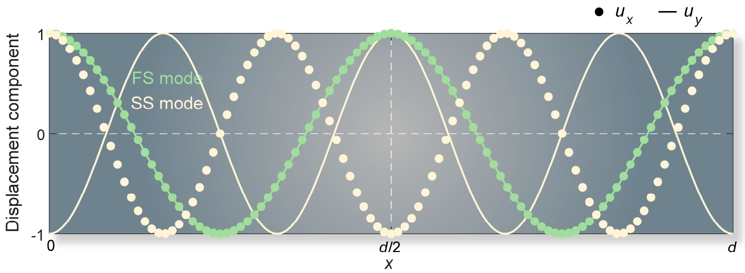

FIG. S3 The normalized displacement components of the FS and SS modes at f=$2f_{MC}$ for the TFPI used in Figure 1 of the main text.

1.6 Effect of the unit cell size

The effective properties C_{ij} of the anisotropic TFPI are evaluated by the static homogenization method. Therefore, in the full-wave simulation where the effective properties are actually frequency-dependent, the size (a) of the metamaterial unit cell should be sufficiently smaller than the interferometer width (d). Otherwise the TFPI will not exhibit the effective material properties that are demanded for perfect PMC to occur. FIG S4 presents the transmission curves for a=d/50 and a=d/100 obtained through full-wave simulation. It compares the numerical full-wave simulation results with the analytical results. While the numerical results do not agree very well with the analytic results for a=d/50 in the high frequency range, they agree well with each other for a=d/100 over all frequencies considered. Therefore, we used a=d/100 to obtain the numerical results in Figure 1c of the main text.

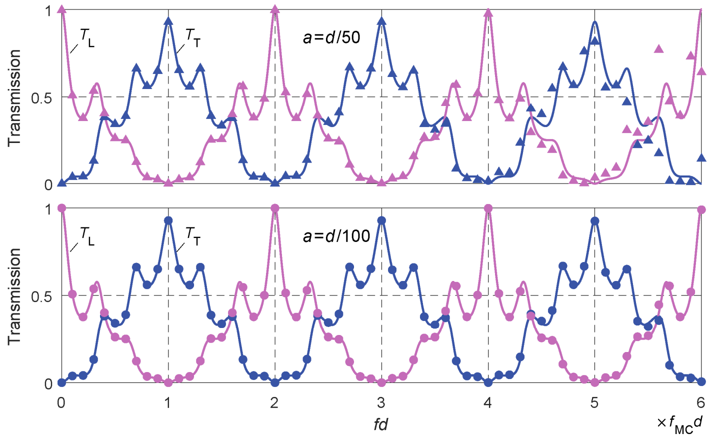

FIG S4 Effect of the unit cell size on the full-wave numerical simulations. Lines: analytical results; triangles: numerical results with $a=d/50$; dots: numerical results with $a=d/100$. All other parameters are the same as used for Figure 1 of the main text.

1.7 Conversion from T wave to L wave

As mentioned earlier, PMC is also valid when a T wave is normally incident. Therefore, a perfect T-to-L mode-conversion phenomenon can also be realized with the same interferometer shown in Figure 1 of the main text. The transmission and reflection power ratios for normally incident T waves are shown in FIG S5a. Note that the T-to-L mode conversion ratio at the PMC frequencies is 92.88%, which is the same as the L-to-T mode conversion ratio.

FIG S5b shows the snapshots of the transient displacement when a harmonic T wave propagates through the TFPI. When the harmonic wave is excited at the PMC frequencies f_{MC} and $3f_{MC}$, the incident T wave is perfectly converted to an L wave. When the harmonic wave is excited at the mode-persevering frequency ($2f_{MC}$), the incident T wave is transmitted without any mode conversion.

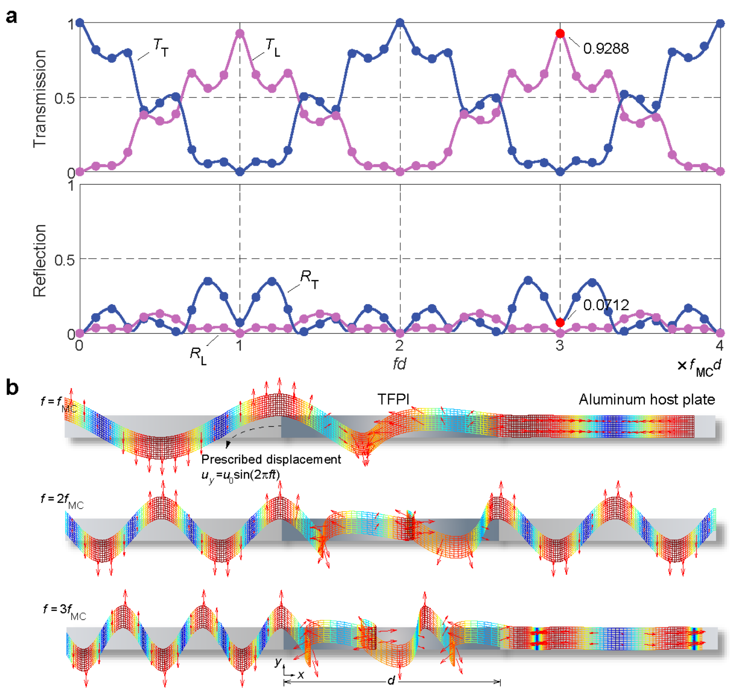

FIG S5 PMC with a normally incident T wave. T_L (a) (T-to-L transmission, in this case) and T_T (T-to-T transmission, in this case) and the reflection ratios R_L (T-to-L reflection, in this case) and R_T (T-to-T reflection, in this case) through the TFPI, calculated by the theoretical analysis (lines) and full-wave numerical simulation (dots). (b) Snapshots of transient displacement for a normally incident harmonic T wave. The prescribed vertical displacement (u_y) at x=0 is $u_0\sin(2\pi ft)$. All the snapshots are captured at t=29.25/f. All parameters are the same as those in Figure 1 of the main text.

1.8 Perfect mode-converting interference with a different set of n_{FS} and n_{SS}

We showed that perfect mode-converting interference occurs if the two sets of conditions stated in Eq. (S42) and Eq. (S43) (i.e. Eq. (S26) and Eq. (S15)) are exactly satisfied. In the main text, the effective properties of the designed TFPI in Figure 1 satisfy Eq. (S15) with n_{FS}=2 and n_{SS}=3; For Figure. 2a of the main text, we also used n_{FS}=2 and n_{SS}=3.

Here, we will investigate PMC corresponding to a different set of n_{FS}=1 and n_{SS}=2. If the material properties of this TFPI have the following values, the PMC conditions are exactly satisfied for the selected n_{FS} and n_{SS} values with $f_{MC}d$=2.45kHz·m:

$$\left(C_{11},C_{66},C_{16}\right)=\left(45,45,27\right)\text{GPa}, \rho=3000\text{kg/m}^3. \tag{S46}$$

The TFPI is assumed to be sandwiched by aluminum.

The transmission and reflection ratios are shown in FIG S6a. The frequency spectra exhibit the same pattern as those shown in Figure 1b and Figure 2a. At the PMC frequencies, T_T is maximized and T_L is identically zero; perfect mode conversion occurs. Note that T_T at the PMC frequencies is determined only by the Poisson's ratio of the base isotropic medium. Therefore, the value of T_T at the PMC frequencies is the same as that shown in Figure 1b and Figure 2a.

FIG S6b shows the snapshots of the transient displacement field, and FIG S7 shows the particle motions inside the TFPI. Because the formation mechanism of PMC for n_{FS}=1 and n_{SS} =2 is found to be the same as that for n_{FS}=2 and n_{SS} =3, no repeated analysis would be necessary with these figures.

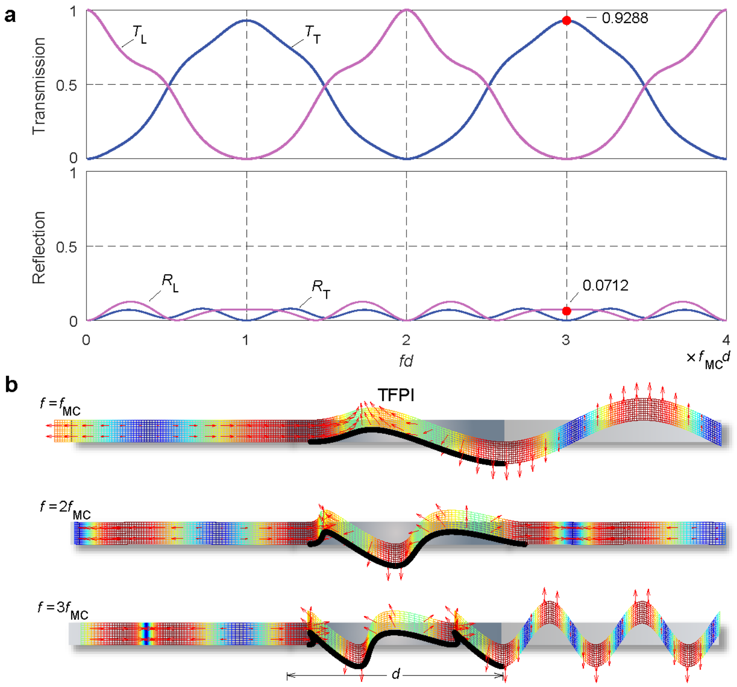

FIG S6 PMC through a TFPI corresponding to a different set of n_{FS} =1 and n_{SS}=2. (a) The transmission and reflection ratios for. An L wave is assumed to be incident to the TFPI sandwiched by the aluminum base medium. $f_{MC}d$=2.45kHz·m. (b) Snapshots of transient displacement for a normally incident harmonic L. The prescribed horizontal displacement (u_x) at x=0 is $u_0\sin(2\pi ft)$. The snapshots are captured at t=29.25/f.

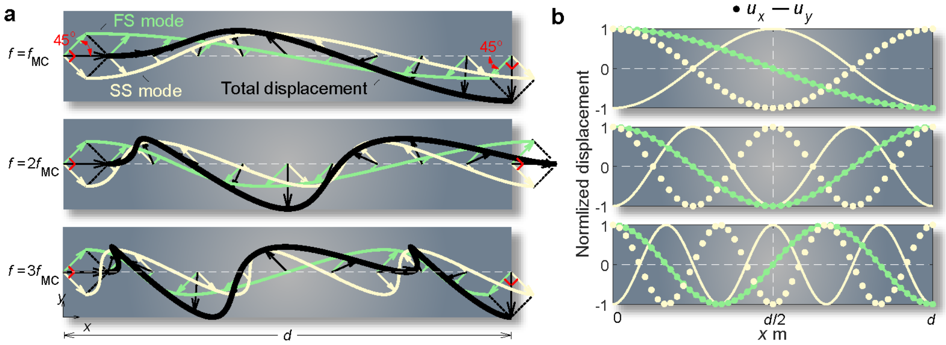

FIG S7 Particle motions inside the interferometer for n_{FS}=1 and n_{SS} =2. (a) Schematic demonstration of the mode conversion mechanism by means of two L-T coupled wave modes inside the interferemeter, denoted as the fast skew mode (FS) and the slow skew mode (SS). (b) Distribution of horizontal (u_x) and vertical (u_y) displacement components of the FS and SS modes inside the interferometer.

2 Design of perfect TFPI

2.1 A design procedure

We showed that perfect mode-converting interference occurs when the two conditions stated by Eqs. (S42) and (S43) are satisfied. Because it is difficult or nearly impossible to find a natural material the stiffness of which satisfies (S42) and (S43) exactly, we realize the interferometer by a metamaterial.

To facilitate the design of the perfect TFPI exhibiting PMC, the condition (S42) (equivalently, (S27)) is expressed as

$$\kappa = 1 \tag{S47}$$

with

$$\kappa \triangleq \frac{2\Gamma n_{\mathrm{FS}} n_{\mathrm{SS}}}{\Pi(n_{\mathrm{FS}}^2 + n_{\mathrm{SS}}^2)} = \frac{n_{\mathrm{FS}} n_{\mathrm{SS}} (C_{11} + C_{66})}{(n_{\mathrm{FS}}^2 + n_{\mathrm{SS}}^2)\sqrt{C_{11}C_{66} - C_{16}^2}}. \tag{S48}$$

Likewise, the condition (S43) is written as

$$\gamma = \frac{C_{11}}{\left(C_{11} + C_{66}\right)/2} = 1. \tag{S49}$$

For the design of the TFPI, therefore, we can check the satisfaction degree of the conditions that κ=1 and γ=1. Since neither κ nor γ involves the density ρ of the anisotropic metamaterial, we only need to determine the values of C_{ij} that can make κ=1 and γ=1 as precisely as possible. After a specific metamaterial satisfying (S47) and (S49) is found, one can simply calculate its mass density ρ. With determined C_{ij} and ρ, we can find $f_{\mathrm{MC}}d$ by solving Eq. (S27) as

$$f_{\mathrm{MC}}d = \sqrt{\frac{\Pi n_{\mathrm{FS}} n_{\mathrm{SS}}}{4\rho}} = \sqrt{\frac{\Gamma n_{\mathrm{FS}}^2 n_{\mathrm{SS}}^2}{2\rho\left(n_{\mathrm{FS}}^2 + n_{\mathrm{SS}}^2\right)}}. \tag{S50}$$

Although $f_{\mathrm{MC}}d$ is not known until an actual perfect TFPI is obtained, one can choose d arbitrarily once f_{MC} is given.

2.2 Practical design

We will explain how the unit cell shown in Figure 1c of the main text was designed. After choosing n_{FS}=2 and n_{SS}=3, void slits are inserted in the unit cell. The unit cell is made of aluminum which is the same material as the base material. After trials and errors, l_2=0.0800a, l_3=0.1200a, l_4=0.1850a, r=0.1000a, and θ=45° were determined. We used the remaining parameter l_1 as a tuning parameter to ensure that κ=1 and γ=1. The unit cell is also shown in FIG S8a. The variations of κ and γ are plotted in FIG S8b and c, respectively. The selected value of l_1/a is marked by a red circle which makes both κ and γ sufficiently close to 1 simultaneously.

For the selected parameter values with l_1=0.2636a, the corresponding effective properties are found to be

$$\left(C_{11}, C_{66}, C_{16}\right) = \left(12.983, 13.053, 5.006\right)\text{GPa}, \rho = 2193\text{kg/m}^3. \qquad \text{(S51)}$$

Because we fabricate the TFPI on a thin host aluminum plate, the plane stress condition was used in evaluating the effective properties. Using Eq.(S50) we found

$$f_{\text{MC}} d = 2.87\text{kHz}\cdot\text{m}. \qquad \text{(S52)}$$

FIG S8d shows the transmission curves for varying l_1 values around the selected value of l_1=0.2636a. The transmission curves are little affected by the variation of l_1 over a wide range of frequencies of interest. However, some errors would occur in the transmission curves if the frequency is high (say, fd>8$f_{\text{MC}}d$).

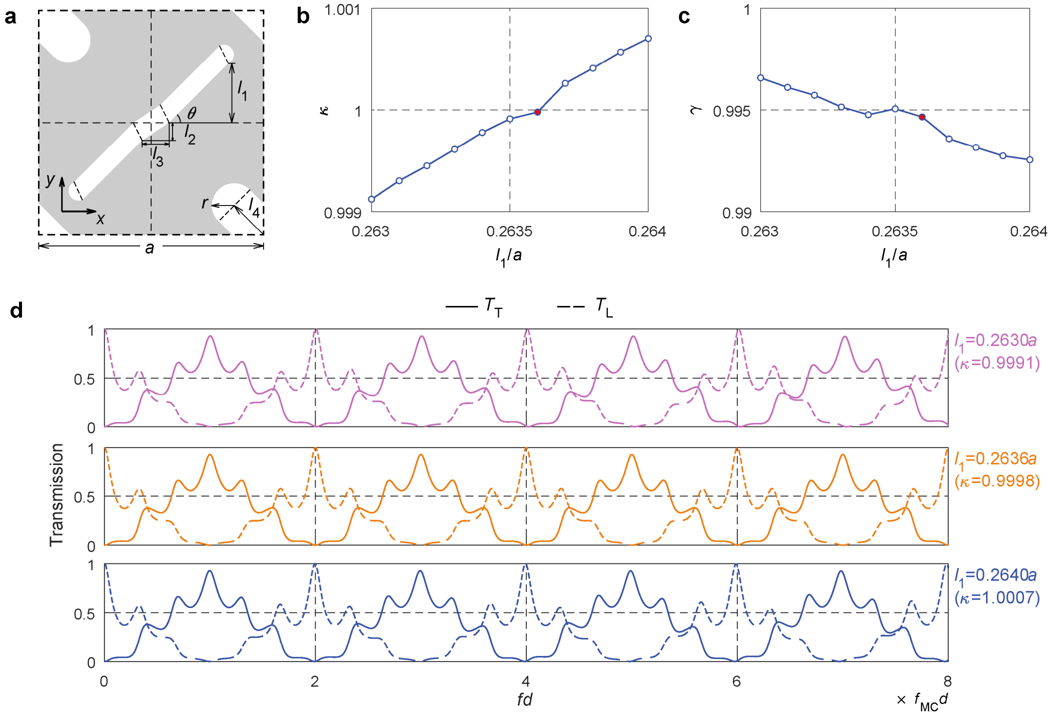

FIG S8. Proposed unit cell of the TFPI (n_{FS}=2,n_{SS}=3). (a) Geometric parameters of the unit cell. (b) The relationship between κ and l_1, when l_2 = 0.04a, l_3 = 0.12a, l_4 = 0.185a, r = 0.1a, and θ=45°. (c) The relationship between γ and l_1. (d) Transmission ratios through anisotropic metaslab with different l_1. $f_{\text{MC}}d$ = 2.87 kHz·m.

3. Experimental details

This section explains the details of the fabrication of the sample and experimental procedure.

The metamaterial unit cells shown in FIG S8a may not be precisely fabricated by machining because the geometry is rather complicated. To facilitate the fabrication, the unit configuration shown in FIG S8a was somewhat modified to the one shown in FIG S9a (also shown in Figure 4 in the main text).

For the modified unit cell shown in FIG S9a, the effective properties can be estimated by the

static homogenization method as,

$$\left(C_{11}, C_{66}, C_{16}\right) = \left(10.683, 11.921, 4.393\right)\text{GPa}, \rho = 1920\text{kg/m}^3. \tag{S53}$$

The corresponding $f_{\text{MC}}d$ is found to be,

$$f_{\text{MC}}d = 2.86\text{kHz}\cdot\text{m}. \tag{S54}$$

which is virtually the same as that of the original one. Although the stiffness values in Eq. (S53) are not exactly the same as those in Eq. (S51), they satisfy the two conditions given by (S15) and (S26) fairly well because the corresponding values of κ and γ are fairly close to 1:

$$\kappa = 1.0036, \gamma = 0.9452. \tag{S55}$$

FIG S9c compares the transmission curves obtained with the original design shown in FIG S8a, and the modified one shown in FIG S9a.The L-to-T transmission power ratio T_{T} at the PMC frequencies for the modified design is a little bit lower than that of the original design and T_{L} is not identically zero. However, the two results are sufficiently close and the modified unit cell clearly shows the PMC phenomena with distinct peaks in the transmission spectra.

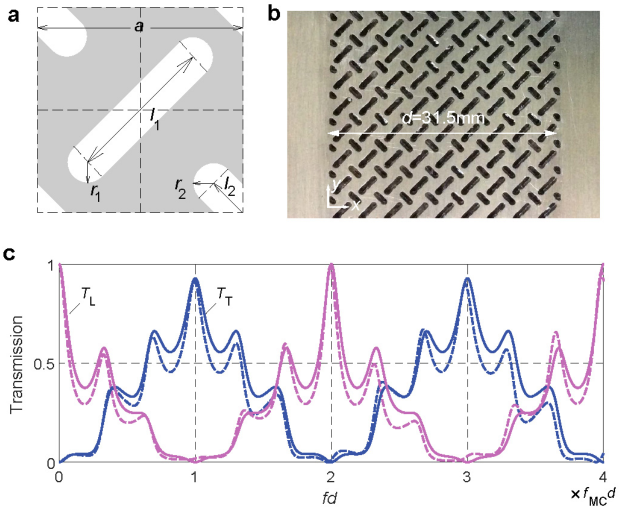

FIG S9. Modified unit cell for actual fabrication of the TFPI (a) the unit cell configuration (l_1= 0.73a, l_2=0.20a, and r_1=r_2=0.1a, a=3.5mm). (b) Photo of the sample (only partly shown). (c) Comparison of the transmission by the original design and the modified one. Solid lines: original design shown in FIG S8a; dashed lines: modified design shown in FIG S9a. The transmission is evaluated analytically using the effective properties. The TFPI is sandwiched by aluminum. $f_{\text{MC}}d$ =2.87kHz·m.

For the actual design of the TFPI, we chose d=31.5mm for its thickness so that f_{MC} can be set at the target frequency. If the unit cell size a is sufficiently small, one can clearly observe the PMC phenomenon over a wide range of frequency, as demonstrated in FIG S4. However, it will be difficult to fabricate with a very small a. For our experiments, the wave experiment around the lowest PMC frequency f=f_{MC} is focused. After some numerical simulations,

a=d/9=3.5mm is found reasonable and actually used for our experiments. The reason behind this is that at f=f_{MC}, d=λ_{FS}=1.5λ_{SS}, which means

$$a=\frac{\lambda_{\mathrm{FS}}}{9}=\frac{\lambda_{\mathrm{SS}}}{6}. \tag{S56}$$

Eq. (S56) indicates that the unit cell is of subwavelength. The photo of the fabricated interferometer is shown in FIG S9b. FIG S10 shows the photos of the experimental setup. As shown in FIG S10a, we used an aluminum plate large enough to minimize the effect of reflected waves from the boundaries.

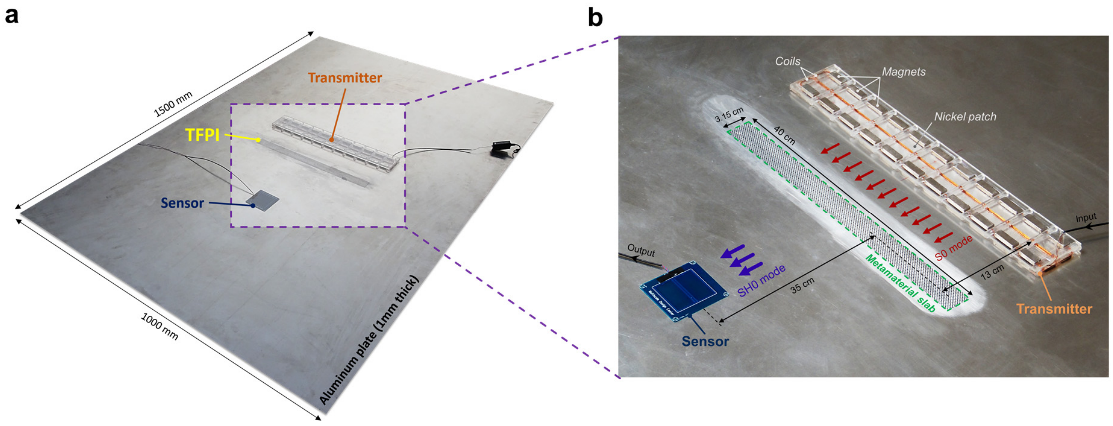

FIG S10. Experimental setup. (a) The whole view of a TFPI-embedded plate waveguide and (b) the zoomed view of the transducers and fabricated metamaterial slab. For experiments, an S0 plane-wave mode generated by a wide MPT (Magnetostrictive Patch Transducer) is to be incident on the TFPI fabricated in a 1 mm thick aluminum plate. At the PMC frequency, the S0 wave mode can be perfectly converted to an SH0 wave mode after passing through the interferometer.

4. Potential applications

4.1 Sound transmission suppression

A promising application would be to minimize acoustic transmission through a panel. Because acoustic waves in fluids (air) can transmit longitudinal (or pressure) waves only, we can minimize acoustic wave transmission through a TFPI by converting a longitudinal (pressure) wave to a transverse wave. It is well known that transverse (shear) waves cannot be transmitted in air.

FIG S11 shows an illustrative simulation result (under the plane stress condition). For the simulation in FIG S11, the elastic medium was assumed to be made of epoxy (E_0=3.6GPa, ρ_0=1180 kg/m^3 and υ_0=0.33). The configuration of the unit cell of the TFPI is the same as that given in FIG S9a. The effective properties of the TFPI are found to be (C_{11}, C_{66}, C_{16}) = (0.620, 0.650, 0.242) GPa and ρ = 841 kg/m^3. For the selected TFPI, one can show that κ and γ are

sufficiently close to 1:

$$\kappa = 0.9985, \gamma = 0.9761 \text{ with } n_{FS} = 2, n_{SS} = 3. \tag{S57}$$

We can also find that $f_{MC}d$ =1.03 kHz·m. The fundamental PMC frequency f_{MC} can be set to be f_{MC}≈10 kHz by choosing d=0.1m.

As illustrated in FIG S11a, a speaker generating acoustic pressure of unit magnitude is placed just in front of the left sides of the panel. We calculate the wave field in air located on the right side of the panel and then compute the transmission loss (*TL*). FIG S11b compares the TL around $f= f_{MC}$. Although the TFPI is effective over a narrow frequency range, the increase in the *TL* becomes as much as 112 dB. FIG S11c shows the displacement field inside the TFPI at $f= f_{MC}$ =10kHz. It shows that the incident longitudinal wave is converted to a transverse wave involving shearing deformation. Therefore, the incident acoustic wave can hardly propagate into the adjacent air located on the right side of the interferometer.

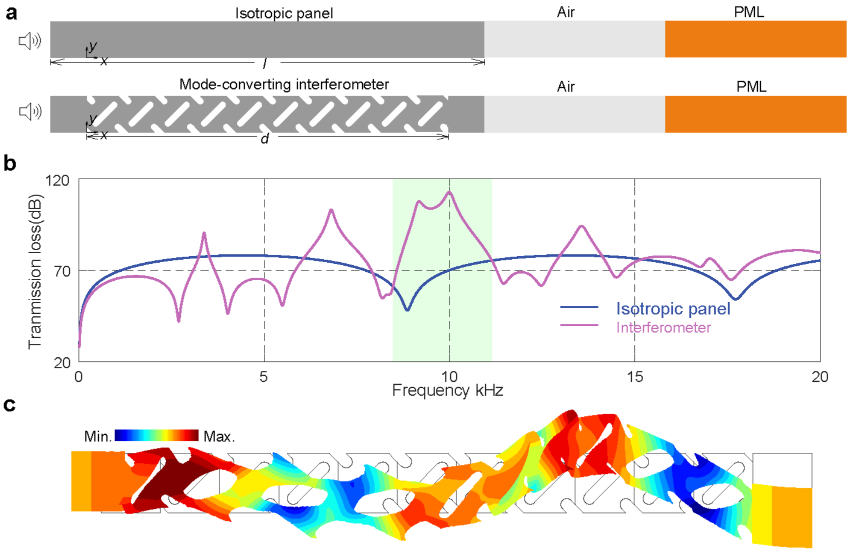

FIG S11. Application of the perfect mode-conversion phenomenon for sound transmission suppression. (a) Simulation models. The upper and lower boundaries are defined to be Floquet periodic. Acoustic pressure of 1 Pa amplitude is applied on the left boundary of the system. d=0.12m, l=0.1m. (b) Comparison of the transmission curves for the isotropic panel and the TFPI around at the PMC frequency f_{MC}= 10 kHz. (c) Illustration of the displacement field inside the TFPI at f=f_{MC}.

4.2 Efficient wave generation of transverse (or shear) waves

A method using Snell's critical angle is a traditional method to convert L waves to T waves. Because the direct generation of high-power T waves is not easy, T waves are typically generated by mode conversion from L waves that are much easier to generate by available (and widely used) piezoelectric transducers. As T waves have received much attention in medical applications where ultrasound waves are to be delivered to the human brain through its skull, efficient T-wave generation by PMC could be critically useful. Also, the T-wave mode is used

in ultrasound-based flow meters. In either case, a wedge based on Snell's critical angle, typically made of acrylic resin, is needed, but large impedance mismatch between the wedge and a test object hinders efficient power transmission Typically, the mode-conversion transmission efficiency by the wedge approach is 20~30% [20]. Note that the TFPI, which can exactly satisfy the PMC conditions derived in this work, is shown to convert an incident L wave to a T wave with as high as 92.88% mode-conversion transmission for an aluminum base material. (The related results are presented in Figure 1b of the main text.)

Appendix

A.1 M and N matrices,

$$\mathbf{M}=\begin{bmatrix} -\mathrm{j}\omega P_x & -\mathrm{j}\omega P_x & -\mathrm{j}\omega Q_x & -\mathrm{j}\omega Q_x \\ -\mathrm{j}\omega P_y & -\mathrm{j}\omega P_y & -\mathrm{j}\omega Q_y & -\mathrm{j}\omega Q_y \\ \mathrm{j}\alpha\left(C_{11}P_x+C_{16}P_y\right) & -\mathrm{j}\alpha\left(C_{11}P_x+C_{16}P_y\right) & \mathrm{j}\beta\left(C_{11}Q_x+C_{16}Q_y\right) & -\mathrm{j}\beta\left(C_{11}Q_x+C_{16}Q_y\right) \\ \mathrm{j}\alpha\left(C_{16}P_x+C_{66}P_y\right) & -\mathrm{j}\alpha\left(C_{16}P_x+C_{66}P_y\right) & \mathrm{j}\beta\left(C_{16}Q_x+C_{66}Q_y\right) & -\mathrm{j}\beta\left(C_{16}Q_x+C_{66}Q_y\right) \end{bmatrix} \tag{S58}$$

$$\mathbf{N}=\begin{bmatrix} \mathrm{e}^{\mathrm{j}\alpha x} & & & \\ & \mathrm{e}^{-\mathrm{j}\alpha x} & & \\ & & \mathrm{e}^{\mathrm{j}\beta x} & \\ & & & \mathrm{e}^{-\mathrm{j}\beta x} \end{bmatrix}. \tag{S59}$$

A.2 T matrix

$$T_{11}=\frac{P_xQ_y\cos(\alpha d)-P_yQ_x\cos(\beta d)}{P_xQ_y-P_yQ_x} \tag{S60}$$

$$T_{12}=-\frac{P_xQ_x}{P_xQ_y-P_yQ_x}\left[\cos(\alpha d)-\cos(\beta d)\right] \tag{S61}$$

$$T_{13}=\frac{-\mathrm{j}\omega}{\alpha\beta\left(C_{11}C_{66}-C_{16}^2\right)\left(P_xQ_y-P_yQ_x\right)}\left[\beta P_x\left(C_{16}Q_x+C_{66}Q_y\right)\sin(\alpha d)-\alpha Q_x\left(C_{16}P_x+C_{66}P_y\right)\sin(\beta d)\right] \tag{S62}$$

$$T_{14}=\frac{\mathrm{j}\omega}{\alpha\beta\left(C_{11}C_{66}-C_{16}^2\right)\left(P_xQ_y-P_yQ_x\right)}\left[\beta P_x\left(C_{11}Q_x+C_{16}Q_y\right)\sin(\alpha d)-\alpha Q_x\left(C_{11}P_x+C_{16}P_y\right)\sin(\beta d)\right] \tag{S63}$$

$$T_{21}=\frac{P_yQ_y}{P_xQ_y-P_yQ_x}\left[\cos(\alpha d)-\cos(\beta d)\right] \tag{S64}$$

$$T_{22}=-\frac{P_yQ_x\cos(\alpha d)-P_xQ_y\cos(\beta d)}{P_xQ_y-P_yQ_x} \tag{S65}$$

$$T_{23}=\frac{-\mathrm{j}\omega}{\alpha\beta\left(C_{11}C_{66}-C_{16}^2\right)\left(P_xQ_y-P_yQ_x\right)}\left[\beta P_y\left(C_{16}Q_x+C_{66}Q_y\right)\sin(\alpha d)-\alpha Q_y\left(C_{16}P_x+C_{66}P_y\right)\sin(\beta d)\right] \tag{S66}$$

$$T_{24}=\frac{\mathrm{j}\omega}{\alpha\beta\left(C_{11}C_{66}-C_{16}^2\right)\left(P_xQ_y-P_yQ_x\right)}\left[\beta P_y\left(C_{11}Q_x+C_{16}Q_y\right)\sin(\alpha d)-\alpha Q_y\left(C_{11}P_x+C_{16}P_y\right)\sin(\beta d)\right] \tag{S67}$$

$$T_{31}=\frac{-\mathrm{j}}{\omega\left(P_xQ_y-P_yQ_x\right)}\left[\alpha Q_y\left(C_{11}P_x+C_{16}P_y\right)\sin(\alpha d)-\beta P_y\left(C_{11}Q_x+C_{16}Q_y\right)\sin(\beta d)\right] \tag{S68}$$

$$T_{32}=\frac{\mathrm{j}}{\omega\left(P_xQ_y-P_yQ_x\right)}\left[\alpha Q_x\left(C_{11}P_x+C_{16}P_y\right)\sin\left(\alpha d\right)-\beta P_x\left(C_{11}Q_x+C_{16}Q_y\right)\sin\left(\beta d\right)\right] \tag{S69}$$

$$T_{33}=\frac{\left(C_{11}P_x+C_{16}P_y\right)\left(C_{16}Q_x+C_{66}Q_y\right)\cos\left(\alpha d\right)-\left(C_{11}Q_x+C_{16}Q_y\right)\left(C_{16}P_x+C_{66}P_y\right)\cos\left(\beta d\right)}{\left(C_{11}C_{66}-C_{16}^2\right)\left(P_xQ_y-P_yQ_x\right)} \tag{S70}$$

$$T_{34}=-\frac{\left(C_{11}P_x+C_{16}P_y\right)\left(C_{11}Q_x+C_{16}Q_y\right)}{\left(C_{11}C_{66}-C_{16}^2\right)\left(P_xQ_y-P_yQ_x\right)}\left[\cos\left(\alpha d\right)-\cos\left(\beta d\right)\right] \tag{S71}$$

$$T_{41}=\frac{-\mathrm{j}}{\omega\left(P_xQ_y-P_yQ_x\right)}\left[\alpha Q_y\left(C_{16}P_x+C_{66}P_y\right)\sin\left(\alpha d\right)-\beta P_y\left(C_{16}Q_x+C_{66}Q_y\right)\sin\left(\beta d\right)\right] \tag{S72}$$

$$T_{42}=\frac{\mathrm{j}}{\omega\left(P_xQ_y-P_yQ_x\right)}\left[\alpha Q_x\left(C_{16}P_x+C_{66}P_y\right)\sin\left(\alpha d\right)-\beta P_x\left(C_{16}Q_x+C_{66}Q_y\right)\sin\left(\beta d\right)\right] \tag{S73}$$

$$T_{43}=\frac{\left(C_{16}P_x+C_{66}P_y\right)\left(C_{16}Q_x+C_{66}Q_y\right)}{\left(C_{11}C_{66}-C_{16}^2\right)\left(P_xQ_y-P_yQ_x\right)}\left[\cos\left(\alpha d\right)-\cos\left(\beta d\right)\right] \tag{S74}$$

$$T_{44}=-\frac{\left(C_{16}P_x+C_{66}P_y\right)\left(C_{11}Q_x+C_{16}Q_y\right)\cos\left(\alpha d\right)-\left(C_{16}Q_x+C_{66}Q_y\right)\left(C_{11}P_x+C_{16}P_y\right)\cos\left(\beta d\right)}{\left(C_{11}C_{66}-C_{16}^2\right)\left(P_xQ_y-P_yQ_x\right)} \tag{S75}$$

A.3 S matrix

$$S_{11}=\frac{1}{2}T_{11}-\frac{1}{2}\frac{\omega}{\alpha_0c_{11}}T_{31}-\frac{1}{2}\frac{\alpha_0c_{11}}{\omega}T_{13}+\frac{1}{2}T_{33} \tag{S76}$$

$$S_{12}=\frac{1}{2}T_{11}-\frac{1}{2}\frac{\omega}{\alpha_0c_{11}}T_{31}+\frac{1}{2}\frac{\alpha_0c_{11}}{\omega}T_{13}-\frac{1}{2}T_{33} \tag{S77}$$

$$S_{13}=\frac{1}{2}T_{12}-\frac{1}{2}\frac{\omega}{\alpha_0c_{11}}T_{32}-\frac{1}{2}\frac{\beta_0c_{66}}{\omega}T_{14}+\frac{1}{2}\frac{\beta_0c_{66}}{\alpha_0c_{11}}T_{34} \tag{S78}$$

$$S_{14}=\frac{1}{2}T_{12}-\frac{1}{2}\frac{\omega}{\alpha_0c_{11}}T_{32}+\frac{1}{2}\frac{\beta_0c_{66}}{\omega}T_{14}-\frac{1}{2}\frac{\beta_0c_{66}}{\alpha_0c_{11}}T_{34} \tag{S79}$$

$$S_{21}=\frac{1}{2}T_{11}+\frac{1}{2}\frac{\omega}{\alpha_0c_{11}}T_{31}-\frac{1}{2}\frac{\alpha_0c_{11}}{\omega}T_{13}-\frac{1}{2}T_{33} \tag{S80}$$

$$S_{22}=\frac{1}{2}T_{11}+\frac{1}{2}\frac{\omega}{\alpha_0c_{11}}T_{31}+\frac{1}{2}\frac{\alpha_0c_{11}}{\omega}T_{13}+\frac{1}{2}T_{33} \tag{S81}$$

$$S_{23}=\frac{1}{2}T_{12}+\frac{1}{2}\frac{\omega}{\alpha_0c_{11}}T_{32}-\frac{1}{2}\frac{\beta_0c_{66}}{\omega}T_{14}-\frac{1}{2}\frac{\beta_0c_{66}}{\alpha_0c_{11}}T_{34} \tag{S82}$$

$$S_{24}=\frac{1}{2}T_{12}+\frac{1}{2}\frac{\omega}{\alpha_0c_{11}}T_{32}+\frac{1}{2}\frac{\beta_0c_{66}}{\omega}T_{14}+\frac{1}{2}\frac{\beta c_{66}}{\alpha_0c_{11}}T_{34} \tag{S83}$$

$$S_{31}=\frac{1}{2}T_{21}-\frac{1}{2}\frac{\omega}{\beta_0c_{66}}T_{41}-\frac{1}{2}\frac{\alpha_0c_{11}}{\omega}T_{23}+\frac{1}{2}\frac{\alpha_0c_{11}}{\beta_0c_{66}}T_{43} \tag{S84}$$

$$S_{32}=\frac{1}{2}T_{21}-\frac{1}{2}\frac{\omega}{\beta_0c_{66}}T_{41}+\frac{1}{2}\frac{\alpha_0c_{11}}{\omega}T_{23}-\frac{1}{2}\frac{\alpha_0c_{11}}{\beta_0c_{66}}T_{43} \tag{S85}$$

$$S_{33}=\frac{1}{2}T_{22}-\frac{1}{2}\frac{\omega}{\beta_0c_{66}}T_{42}-\frac{1}{2}\frac{\beta_0c_{66}}{\omega}T_{24}+\frac{1}{2}T_{44} \tag{S86}$$

$$S_{34}=\frac{1}{2}T_{22}-\frac{1}{2}\frac{\omega}{\beta_0c_{66}}T_{42}+\frac{1}{2}\frac{\beta_0c_{66}}{\omega}T_{24}-\frac{1}{2}T_{44} \tag{S87}$$

$$S_{41} = \frac{1}{2}T_{21} + \frac{1}{2}\frac{\omega}{\beta_0 c_{66}}T_{41} - \frac{1}{2}\frac{\alpha_0 c_{11}}{\omega}T_{23} - \frac{1}{2}\frac{\alpha_0 c_{11}}{\beta_0 c_{66}}T_{43} \quad \text{(S88)}$$

$$S_{42} = \frac{1}{2}T_{21} + \frac{1}{2}\frac{\omega}{\beta_0 c_{66}}T_{41} + \frac{1}{2}\frac{\alpha_0 c_{11}}{\omega}T_{23} + \frac{1}{2}\frac{\alpha_0 c_{11}}{\beta_0 c_{66}}T_{43} \quad \text{(S89)}$$

$$S_{43} = \frac{1}{2}T_{22} + \frac{1}{2}\frac{\omega}{\beta_0 c_{66}}T_{42} - \frac{1}{2}\frac{\beta_0 c_{66}}{\omega}T_{24} - \frac{1}{2}T_{44} \quad \text{(S90)}$$

$$S_{44} = \frac{1}{2}T_{22} + \frac{1}{2}\frac{\omega}{\beta_0 c_{66}}T_{42} + \frac{1}{2}\frac{\beta_0 c_{66}}{\omega}T_{24} + \frac{1}{2}T_{44} \quad \text{(S91)}$$

References

S1. Tsvankin., I. Anisotropic parameters and P-wave velocity for orthorhombic media. *Geophysics*, **62** 1292, (1997).
